# A case–control study investigating food addiction in Parkinson patients

**DOI:** 10.1038/s41598-021-90266-8

**Published:** 2021-05-25

**Authors:** Ingrid de Chazeron, F. Durif, C. Lambert, I. Chereau-Boudet, M. L. Fantini, A. Marques, P. Derost, B. Debilly, G. Brousse, Y. Boirie, P. M. Llorca

**Affiliations:** 1 Service de Psychiatrie B, CHU Clermont-Ferrand, EA7280, Université Clermont Auvergne, Rue Montalembert BP 69, 63003 Clermont-Ferrand, France; 2 Service de Neurologie A, CHU Clermont-Ferrand, EA7280, Université Clermont Auvergne, Clermont-Ferrand, France; 3 Unité de Biostatistiques (DRCI), CHU Clermont-Ferrand, Clermont-Ferrand, France; 4 Explorations Fonctionnelles du Système Nerveux, CHU Clermont-Ferrand, EA7280, Université Clermont Auvergne,, Clermont-Ferrand, France; 5 Service de Nutrition Clinique, INRA, UMR 1019, UNH, CRNH Auvergne, CHU Clermont-Ferrand, Clermont-Ferrand, France; 6 Centre Référent Troubles Des Conduites Alimentaires, CHU Clermont-Ferrand, Clermont-Ferrand, France

**Keywords:** Neurology, Risk factors

## Abstract

Eating disorders (EDs) in patients with Parkinson’s disease (PD) are mainly described through impulse control disorders but represent one end of the spectrum of food addiction (FA). Although not formally recognized by DSM-5, FA is well described in the literature on animal models and humans, but data on prevalence and risk factors compared with healthy controls (HCs) are lacking. We conducted a cross-sectional study including 200 patients with PD and 200 age- and gender-matched HCs. Characteristics including clinical data (features of PD/current medication) were collected. FA was rated using DSM-5 criteria and the Questionnaire on Eating and Weight Patterns-Revised (QEWP-R). Patients with PD had more EDs compared to HCs (27.0% vs. 13.0%, respectively, *p* < 0.001). They mainly had FA (24.5% vs. 12.0%, *p* = 0.001) and night eating syndrome (7.0% vs. 2.5% *p* = 0.03). In PD patients, FA was associated with female gender (*p* = 0.04) and impulsivity (higher attentional non-planning factor) but not with the dose or class of dopaminergic therapy. Vigilance is necessary, especially for PD women and in patients with specific impulsive personality traits. Counterintuitively, agonist dopaminergic treatment should not be used as an indication for screening FA in patients with PD.

## Introduction

In Parkinson’s disease (PD), compulsive eating is part of the spectrum of impulse control disorders (ICDs) that also include pathologic gambling, compulsive buying and hypersexuality^1^. Eating disorders (EDs) are abnormal eating habits that negatively affect a person's physical and/or mental health ^2^. The psychiatric community categorizes these disorders as separate entities with a distinct set of symptoms [i.e. binge eating disorder (BED)] but the general term may also include food addiction (FA). The concept of FA, a highly controversial subject, refers to people who exhibit substance dependence symptoms in relation to certain high-fat and high-sugar foods^3^.

The prevalence of EDs (e.g. binge eating disorder or compulsive eating) in patients with PD varies greatly from 0.32 to 11.13%^4,5^. This wide range reflects the various methods (e.g., telephone interviews, patient self-reports, retrospective database research regarding psychological scales, etc.) and tools used, but it also reflects the variability of ED symptomatology. Compulsive eating is characterized by the uncontrollable consumption of a larger amount of food than usual, in excess of that necessary to alleviate hunger. We previously observed that the definition of EDs should include symptoms of food addiction (FA) corresponding to a diminution of control over consumption, which appears to be a core feature^6^. Furthermore, whether or not binge eating disorder (compulsive overeating, BED) points to an addiction remains unclear^7^.

In PD, a relationship between EDs and dopaminergic treatment, particularly dopamine agonists, has been mainly suspected, although the combined influence of various factors^8^ has been mentioned. As a result, it is necessary to identify the precise underlying neurobiological substrates^9^. Therefore, even if new animal models of ICDs are being implemented, it would also be of interest to investigate these behaviors in patients without PD at the same ages. However, little is known regarding the prevalence and symptomatology of FA in the general elderly population. We do not have data on FA or the age of disease onset for the general population. A recent cross-national community survey revealed a median age-of-onset in the late teens to early 20 s and a persistence of 4.3 years^10^ for a specific pathology such as BED. Like in many other investigations, the representation of subjects ≥ 60 years old was weaker and could not correctly describe this phenomenon in this population.

Previous work suggests that BED is particularly prevalent in older adults, and the prevalence of all eating disorders was around 3.5% in older women and 1–2% in older men^11,12^. Indeed, people with EDs can experience varied symptoms, ranging from BED to bulimia nervosa or other specific feeding and eating disorders (OSFED) that have been included in recent modifications of DSM-5^2^ in order to better represent their behaviors and symptoms. These behaviors and symptoms can also be embedded in FA. The new nosological entity of FA^13^ could be of interest when investigating EDs in patients with PD. FA is a clinical and multidimensional concept. Gearhardt et al.^14^ proposed the concept, confirmed by Gordon et al.^3^, of FA as a specific phenotype defined by applying the diagnostic criteria for substance dependence to particular processed foods with added sweeteners or fats. FA was found to be more prevalent not only in obese individuals^15^ but also in patients with alterations in brain circuitry similar to those found in drug addiction^16^.

Some studies have already investigated the relationship between FA and BED such as their overlaps and possible unique differences, although this has generally been done in specific populations like obese participants^17^.

The aim of this study was to explore the prevalence, clinical spectrum and personality profile correlates of FA in a group of subjects with PD compared with age- and gender-matched healthy controls (HCs).

## Methods

### Participants

Patients with PD (based on the UK Brain Bank criteria) were screened during regularly scheduled follow-up visits at the Movement Disorders Unit of Clermont-Ferrand Hospital, France, over a 12-month period. HCs were recruited at health check centers in the same region over a 24-month period.

Exclusion criteria were chronic illness (with the exception of PD for the PD group and no significant neurological dysfunction for the HC group) that could influence eating habits (e.g., diabetes) and inability to understand the instructions (language or cognitive impairment based on the Mini-Mental State Examination < 24). HCs were matched (1:1) for age (± 4 years) and gender and all had no prior history of a neurological disorder. Initially, 220 subjects with PD and 230 HCs were recruited, 20 were excluded in the PD group and 30 in the HC group. Subjects were treated under routine clinical care. The STROBE guidelines^18^ were used to ensure the reporting of this paired study. All the methods used for this study were carried out in accordance with relevant guidelines and regulations. All the procedures performed in the studies involving human participants were in accordance with the ethical standards of the institutional and/or national research committee. The study was approved by the French Ethic Committee. It has been qualified as an observational study and need of informed consent was waived by this Committees of Protection of Persons South-East 6 pursuant to L.1121-1-1 and R.1121-3 of the French Public Health Code in force at the time of the research.

### Study outcomes

Sociodemographic and anthropometric parameters (height and weight) and clinical data (features of PD), including medical details, were collected. On the same day, patients with PD underwent the following assessment procedures: PD medical history and current medication were collected through patient interviews and confirmed with medical records. Anti-Parkinson medications were converted into a levodopa equivalent daily dose (LEDD)^19^. Subjects were then administered sections of the Movement Disorders Society Unified Parkinson Disease Rating Scale (MDS-UPDRS), which includes an interview and examination by a movement disorder neurologist. Subscores for non-motor experiences of daily living (part I) and motor examination (part III) of the MDS-UPDRS were calculated.

We assessed neuropsychiatric symptoms, such as punding, compulsive buying, pathological gambling and hypersexuality using questions derived from the standardized interview “Ardouin Scale of Behavior in Parkinson’s Disease” (ASBPD)^20^.

We assessed psychological distress with the Hospital Anxiety and Depression Scale (HADS) in patients with PD and HCs^21^. HADS is a self-assessment questionnaire that has been found to be a reliable instrument for detecting states of anxiety and depression; seven items are related to depression and seven to anxiety subscales. Scoring for each item ranges from zero to three, with three denoting the highest anxiety or depression level. A total subscale score of > 11 points out of a possible 21 denotes considerable symptoms of anxiety or depression.

Impulsivity was assessed with the Barratt impulsiveness scale (BIS-11)^22^, which is a 30-item questionnaire designed to assess common impulsive behaviors in three separable dimensions: attentional impulsiveness (defined as the [in-]ability to concentrate or focus attention), motor impulsiveness (tendency to act without thinking), and non-planning impulsiveness (lack of future planning and forethought). BIS-11 is composed of 30 items scored on a Likert scale (ranging from never = 1 point to very frequently = 4 points). The total score is the sum of all the items and it also assesses the three main dimensions of impulsive behavior: attentional (11 items), motor (11 items), and non-planning impulsivity (8 items).

Following the interview and examination, the participants were asked to provide individual information regarding food habits and EDs, including FA (DSM-5 criteria and the Questionnaire on Eating and Weight Patterns-Revised [QEWP-R])^23^ and food cravings^24^.

The food craving questionnaire is composed of 14 questions used in a general evaluation study^24^. Three items scored on a Likert scale (ranging from 0 to 4 points rated proportionally to frequency or intensity). One item measures alteration of their daily rhythm of life to obtain a particular food (binary data). We used it only for the description of food craving in relation to a dimension already well known in other specific questionnaires like the FCQ (intense desire to eat…).

The QEWP-R primarily focuses on assessing diagnostic criteria for BED but also assesses night eating syndrome (NES) and includes clinically significant impairment or distress, criteria needed for FA diagnosis. QEWP-R is a 28-item self-report instrument. It is designed to assess the components, duration, and frequency requirements for BED diagnosis.

The Yale Food Addiction Scale (YFAS)^25^ is currently the best available measure for evaluating food addiction, but a variety of approaches have also been used to measure FA including self-report questionnaires^26^ and patient self-identification^27^. This study was conducted during a routine regular visit. We usually use psychiatric and neurological assessments in the ward but were unable to introduce a new tool, so we decided to use only 2 items from the DSM-5 criteria for FA symptoms: 1/Food often consumed in larger amounts or over a longer period or more frequently than was intended, and 2/Craving, or a strong desire or urge to eat specific foods. These items have already been empirically supported in the concept of FA^28^. FA is diagnosed when the participant reports two symptoms (DSM-5) plus clinically significant impairment/distress (QEWP-R). This scoring is in accordance with YFAS2.0^29^.

NES is a circadian delay in the pattern of daily food intake, with evening hyperphagia and/or nocturnal ingestions of food. The term EDs in this paper is used to include FA and more characterized eating troubles, such as BED, NES, purging bulimia nervosa, non-purging bulimia nervosa, etc. The quality of food that induces craving has been classified by the FDA and Drewnowski^30^ into two categories regarding sugar-fat ratio.

### Statistical methods

Statistical analysis was performed using Stata software (StataCorp. 2015. Stata Statistical Software: Release 14. College Station, TX: StataCorp LP). The tests were two-sided, with a type I error set at α = 0.05. Categorical parameters were expressed as frequencies and associated percentages, and continuous data as means and standard deviation (SDs) or as medians and interquartile ranges (IQRs), according to statistical distribution. The prevalence of FA was presented with a 95% confidence interval (CI). Categorical variables were compared between independent groups (PD vs. HC or subjects with vs. without FA) using the chi-squared test or Fisher’s exact test. Quantitative data were compared between groups with Student’s t-test or with the Mann–Whitney test, as appropriate. The Gaussian distribution was verified by the Shapiro–Wilk test, and homoscedasticity was verified by the Fisher–Snedecor test. In the context of multivariate analysis, generalized linear mixed models with the logit link function were used, considering the pair (because each patient with PD was matched with an HC) as a random effect. The dependent variable of the models was binary (FA yes or no), and the independent variables were selected according to univariate results and clinical relevance. The results were expressed as odds ratios (ORs) and 95% CIs.

## Results

There were 200 subjects with PD and 200 HCs. The distribution by age and gender was similar between groups due to the matched design. There were 60% men (n = 120) and the mean age was 67.5 years old (SD 9.9). The characteristics of patients with PD and HCs are presented in Table 1. There was no difference between groups for occupational situation, even before being retired. No between-group difference was observed for body mass index (BMI), even looking separately by gender subgroup, but there were significantly more patients with PD with an underweight BMI class compared to HCs and significantly more HCs in in the obesity BMI class than PD subjects. The mean age at onset of PD was 60.2 years (SD 10.0), and the median disease duration was 5.8 years (IQR: 3.0; 10.7). The mean MDS-UPDRS for the medication score for parts I (non-motor experiences of daily living) and III (motor examination) were 1.7 (SD 1.8) and 16.7 (SD 9.8), respectively. Four patients with PD received no dopaminergic treatment, and 173 received levodopa (LEDD 583 mg/day, IQR: 350; 910) either as monotherapy (54 cases) or in association with other medications (49.1% received dopamine agonist [DA] medication). Regarding the last 23 PD patients (LEDD 232 mg/day, IQR: 100; 500), 4 had DA alone, 7 monoamine oxidase inhibitors (MAO-I) and 12 an association of DA and MAO-I.

**Table 1:** Description of HCs and patients with PD (n = 400).

	HCs (n = 200)	PD (n = 200)	*p*
**Occupational situation, n (%)**			0.42
Working	31 (15.5)	24 (12.0)	
Retired	156 (78.0)	160 (80.0)	
Disability	10 (5.0)	15 (7.5)	
Looking for a job	3 (1.5)	1 (0.5)	
**Occupational class when working, n (%)**			0.10
Craft trade and firm managers	1/31 (3.2)	4/24 (16.7)	
Upper managerial staff and professionals	7/31 (22.6)	5/24 (20.8)	
Intermediary occupations	5/31 (16.1)	0/24 (0.0)	
Clerks and employees	18/31 (58.1)	15/24 (62.5)	
**BMI (kg/m**^**2**^**), mean (SD)**	25.9 (4.2)	25.1 (4.1)	0.07
**Range, n (%)**			**0.04**
< 18.5 (Underweight)	1 (0.5)	10 (5.0)	
18.5 to < 25.0 (Normal weight)	90 (45.0)	91 (45.5)	
25.0 to < 30.0 (Overweight)	83 (41.5)	80 (40.0)	
≥ 30.0 (Obesity)	26 (13.0)	19 (9.5)	
**Age at PD onset (years), mean (SD)**	–	60.2 (10.0)	–
**Disease duration (years), median [IQR]**	–	5.8 [3.0; 10.7]	–
**MDS-UPDRS on medication, mean (SD)**			
Part I	–	1.7 (1.8)	–
Part III	–	16.7 (9.8)	–
**PD medication**			
Cumulative LEDD (mg/day), median [IQR]	–	500 [257; 850]	–
Levodopa medication alone, n (%)	–	54 (27.0)	–
Levodopa dose (mg/day), median [IQR]	–	375 [83; 600]	–
DA medication, n (%)	–	101 (50.5)	–
DA dose (LEDD, mg/day), median [IQR]	–	35 [0; 210]	–
**Punding, n (%)**	62 (31.0)	54 (27.0)	0.38
**Compulsive buying, n (%)**	5 (2.5)	17 (8.5)	**0.008**
**Pathological gambling, n (%)**	2 (1.0)	7 (3.5)	0.18
**Hypersexuality, n (%)**	3 (1.5)	7 (3.5)	0.20
**HADS**			
HADS-anxiety score, mean (SD)	7.6 (3.8)	8.3 (4.1)	0.11
Anxiety cases, n (%)	41 (20.5)	58 (29.0)	**0.049**
HADS-depression score, mean (SD)	4.5 (3.5)	6.6 (3.9)	**< 0.001**
Depression cases, n (%)	12 (6.0)	30 (15.0)	**0.003**
**BIS-11, mean (SD)**			
Total score	55.0 (8.5)	57.1 (9.4)	**0.02**
Motor factor	17.1 (3.3)	17.2 (3.8)	0.64
Non-planning factor	22.0 (4.2)	23.5 (4.6)	**< 0.001**
Attentional factor	16.0 (3.1)	16.3 (3.6)	0.30

Bold values indicate statistical significance (*p* < 0.05).

*BMI* body mass index, *BIS-11* Barratt impulsiveness scale, *DA medication* dopamine agonist medication, *HADS* hospital anxiety and depression scale, *HCs* healthy controls, *IQR* interquartile range, *LEDD* levodopa equivalent daily dose, *MAO-I* monoamine oxidase inhibitor, *MDS-UPDRS* movement disorder society-unified Parkinson’s disease rating scale, *PD* Parkinson’s disease, *SD* standard deviation.

More cases of compulsive buying were detected in the PD population compared to the HCs (8.5% vs. 2.5%, respectively, *p* = 0.008), but there were no differences for punding, pathological gambling and hypersexuality. Based on the suggested HADS cut‐off values, 29.0% and 15.0% of patients with PD had definite anxiety and depression cases, significantly more than in the HC population (*p* < 0.05). Patients with PD also had a significantly higher HADS-depression score compared to HCs (6.6 [SD 3.9] vs. 4.5 [SD 3.5], respectively, *p* < 0.001). Overall, patients with PD showed a mean BIS‐11 total score and non-planning impulsivity score significantly higher than those in HCs (respectively *p* = 0.02 and *p* < 0.001), although these differences stayed minimal.

### PD vs HCs subjects

Compared to HCs (Table 2), significantly more subjects with PD had changed their food habits (28.0% vs. 18.0% *p* = 0.02), especially in the last 5 years, which corresponded to their disease duration. Indeed, a large majority (89.3%) of patients related their changes in food habits to PD diagnosis. Compared to HCs, subjects with PD ate mostly between meals (51.0% vs. 36.0%), 12.5% of PD subjects ate between meals during night and day. There were significantly two-fold more EDs in the population with PD compared to HCs (27.0% vs. 13.0%, *p* < 0.001). EDs were largely represented by FA cases (plus one case of purging bulimia nervosa and two cases of BED) (prevalence of FA in patients with PD: 24.5% [95% CI 18.7, 31.1] vs. HCs 12.0% [95% CI 7.8, 17.4]). NES was significantly more frequent in the PD group compared to the HC group (7.0% vs. 2.5%, *p* = 0.03). Regarding food craving, there were many symptomatologic differences between cases of ED in patients with PD or HCs, including frequency of intense desire to eat and strength of desire or difficulty to resist, which were more frequent in the PD group. All cases of EDs that were able to change their daily rhythm of life to satisfy a particular food craving were patients with PD. The food composition that induced craving was different between the PD and HC groups. Patients with PD preferred a high-sugar diet (81.6% for patients with PD vs. 55.2% for HCs, *p* = 0.01) rather than a high-fat diet (71.4% for patients with PD vs. 79.3% for HCs, *p* = 0.44). Chocolate was largely considered as a craving food for 13.0% of subjects with PD vs. 4.5% for HCs (*p* = 0.003).

**Table 2:** Eating disorders in the HC and PD groups (n = 400).

	HCs (n = 200)	PD (n = 200)	*p*
**Have changes in food habits, n (%)**	36 (18.0)	56 (28.0)	**0.02**
**Time for changes in food habits, n (%)**			**0.03**
Never change	164 (82.0)	144 (72.0)	**0.05**^**†**^
Change in the last 5 years	15 (7.5)	33 (16.5)	**0.02**^**†**^
Change between 5 and 10 years	15 (7.5)	19 (9.5)	0.52^†^
Change more than a decade ago	6 (3.0)	4 (2.0)	0.52^†^
**PD link and changes in food habits, n (%)**			
Before PD diagnosis	–	6/56 (10.7)	–
After PD diagnosis	–	50/56 (89.3)	–
After PD treatment	–	13/56 (23.2)	–
**Actual food regularly consumed, n (%)**			**< 0.001**
During the meal	128 (64.0)	98 (49.0)	**0.006**^**†**^
Between meals, during the day	66 (33.0)	70 (35.0)	0.67^†^
Between meals, during the night	3 (1.5)	7 (3.5)	0.40^†^
Between meals, night and day	3 (1.5)	25 (12.5)	**0.004**^**†**^
**EDs, n (%)**	26 (13.0)	54 (27.0)	**< 0.001**
**Purging bulimia nervosa, n (%)**	0 (0.0)	1 (0.5)	1.00
**Non-purging bulimia nervosa, n (%)**	0 (0.0)	0 (0.0)	–
**BED, n (%)**	0 (0.0)	2 (1.0)	0.50
**FA (∅ BED), n (%)**	24 (12.0)	49 (24.5)	**0.001**
**NES, n (%)**	5 (2.5)	14 (7.0)	**0.03**
**Food craving, n (%)**			
A strong/intense desire to eat more than once a week	22 (11.0)	47 (23.5)	**0.001**
Strength of desire: mild to very strong	24 (12.0)	53 (26.5)	**< 0.001**
Difficulty to resist the desire to eat: hard to impossible to resist	17 (8.5)	39 (19.5)	**0.002**
When they do not have their particular food, capable of altering their daily rhythm of life to get it (i.e., go out to buy it at a store)	0 (0.0)	8 (4.0)	**0.007**
**Food composition inducing craving, n (%)**			
High-sugar	16/29 (55.2)	40/49 (81.6)	**0.01**
High-fat	23/29 (79.3)	35/49 (71.4)	0.44
Chocolate	9 (4.5)	26 (13.0)	**0.003**

Bold values indicate statistical significance (*p* < 0.05).

*BED* binge eating disorder, *FA* food addiction, *HC* healthy controls, *NES* night eating syndrome, *PD* Parkinson’s disease, *EDs* eating disorders, ∅: without.

^†^Post-hoc comparison.

### FA characteristics

We explored the characteristics of FA among the whole population of the study (subjects with PD and HCs) (Table 3). Compared to those without FA, there were significantly more female subjects with FA (53.4% vs. 37.0%, *p* = 0.01), with a higher frequency of PD (67.1% vs. 46.2%, *p* = 0.001). The FA group had a higher cumulative LEDD (372 mg/day IQR [0; 663] vs. 0 [0; 450], *p* = 0.002), a higher levodopa dose (74 mg/day IQR [0; 500] vs. 0 [0; 300], *p* = 0.008), more frequent DA medication (38.4% vs. 22.3%, *p* = 0.004) and a higher DA dose (0 mg/day IQR [0; 160] vs. 0 [0; 0], *p* = 0.007). They were more frequently compulsive buyers (12.3% vs. 4.0%, *p* = 0.009), more anxious (HADS-anxiety cases 37.0% vs. 22.0%, *p* = 0.007) but not more depressed. They did not have a higher total impulsivity score (BIS-11), but when looking at the subscore, they had greater attentional impulsiveness (*p* = 0.006). Food composition (high-sugar or high-fat food) inducing food craving was not specific for the FA group compared to the no-FA group, except for chocolate. When looking at the characteristics of PD patients with and without FA, cumulative LEDD(583 mg/day IQR [372; 755] vs. 500 [241; 850], *p* = 0.47), levodopa dose (375 mg/day IQR [74; 750] vs. 365 [83; 600], *p* = 0.56), presence of DA medication (79.6% vs. 70.9%, *p* = 0.23), DA dose (210 mg/day IQR [129; 225] vs. 200 [120; 300], *p* = 0.2) were not significantly different between PD with or without FA. Compared to no-FA, FA-PD patients were more frequently compulsive buyers (16.3% vs. 6.0%, *p* = 0.024), had a similar non-planning impulsiveness score (*p* = 0.87) but greater attentional impulsiveness (*p* = 0.024).

**Table 3:** Characteristics of food addiction (FA) subjects (n = 400).

	Without FA (n = 327)	With FA (n = 73)	*p*
**Gender, n (% male)**	206 (63.0)	34 (46.6)	**0.01**
**Age (years), mean (SD)**	67.9 (9.8)	65.8 (10.1)	0.10
**BMI (kg/m**^**2**^**), mean (SD)**	25.6 (4.2)	25.2 (3.8)	0.48
**PD diagnosis, n (%)**	151 (46.2)	49 (67.1)	**0.001**
**Cumulative LEDD (mg/day), median [IQR]**	0 [0; 450]	372 [0; 663]	**0.002**
**Levodopa dose (mg/day), median [IQR]**	0 [0; 300]	74 [0; 500]	**0.008**
**DA medication, n (%)**	73 (22.3)	28 (38.4)	**0.004**
**DA dose (LEDD, mg/day), median [IQR]**	0 [0; 0]	0 [0; 160]	**0.007**
**Punding, n (%)**	92 (28.1)	24 (32.9)	0.42
**Compulsive buying, n (%)**	13 (4.0)	9 (12.3)	**0.009**
**Pathological gambling, n (%)**	6 (1.8)	3 (4.1)	0.22
**Hypersexuality, n (%)**	8 (2.4)	2 (2.7)	1.00
**HADS**			
HADS-anxiety score, mean (SD)	7.7 (4.0)	8.9 (3.7)	**0.02**
Anxiety cases, n (%)	72 (22.0)	27 (37.0)	**0.007**
HADS-depression score, mean (SD)	5.6 (3.8)	5.5 (3.8)	0.79
Depression cases, n (%)	34 (10.4)	8 (11.0)	0.89
**BIS-11, mean (SD)**			
Total	55.7 (8.9)	57.7 (9.4)	0.09
Motor	17.1 (3.4)	17.5 (4.0)	0.46
Non-planning	22.7 (4.5)	23.1 (4.2)	0.45
Attentional	16.0 (3.3)	17.2 (3.6)	**0.006**
**Food composition inducing craving, n (%)**			
High-sugar	12/16 (75.0)	44/62 (71.0)	1.00
High-fat	9/16 (56.2)	49/62 (79.0)	0.11
Chocolate	7 (2.1)	28 (38.4)	**< 0.001**

Bold values indicate statistical significance (*p* < 0.05).

*BIS-11* Barratt impulsiveness scale, *BMI* body mass index, *DA medication* dopamine agonist medication, *HADS* hospital anxiety and depression scale, *LEDD* levodopa equivalent daily dose, *PD* Parkinson’s disease, *SD* standard deviation.

### Multivariate analysis

**Figure 1:**
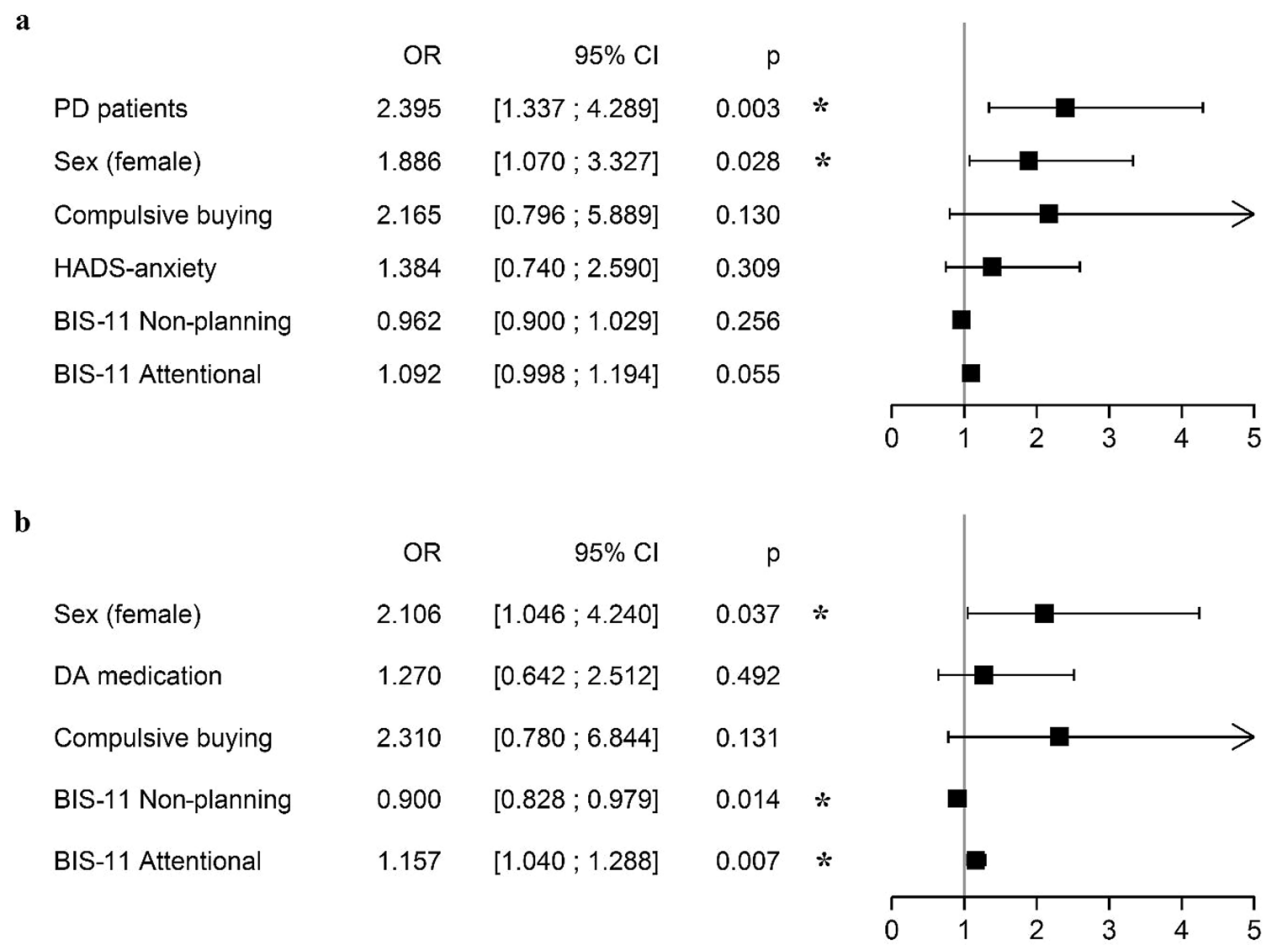
Multivariate analysis of food addiction (FA): a in the whole population (n = 400) and b in PD population (n = 200). *BIS-11 Non-planning* Barratt impulsiveness scale subscore non-planning, *BIS-11 Attentional* Barratt impulsiveness scale subscore attentional, *CI* confidence interval, *DA medication* dopamine agonist medication, *HADS-anxiety* hospital anxiety and depression scale subscore anxiety, *OR* odds ratio, *PD* Parkinson’s disease.

The multivariate analysis was run with significant and clinically relevant factors from the univariate analysis on FA (Fig. 1A). This analysis showed that being a patient with PD (OR 2.40, 95% CI 1.34; 4.29) or a female (OR 1.89, 95% CI 1.07; 3.33) significantly increases the risk of having an FA.

In particular, regarding the population with PD and FA, multivariate analysis (Fig. 1B) showed that being a female (OR 2.11, 95% CI 1.05; 4.24) or having a high attentional impulsiveness score (OR 1.16, 95% CI 1.04; 1.29) increases the risk of having an FA. A high non-planning impulsiveness score (OR 0.90, 95% CI 0.83; 0.98) was protective. The use of DA treatment or being a compulsive buyer did not significantly influence the chances of having an FA in a patient with PD (the DA dose was not significant either, *data not shown*).

## Discussion

In the present study, we investigated EDs in patients with PD compared to a matching general population. Our main findings were: (1) patients with PD had more EDs than the general population; (2) patients with PD had FA and NES, with marginal BED or bulimia; and (3) FA were related to gender and an impulsivity-specific dimension but not to treatment.

To our knowledge, this is the first study specifically investigating EDs in PD compared to a matched paired population with PD. Several studies have consistently shown the presence of EDs in patients with PD, but they generally observed only an extreme fringe of EDs that corresponded to BED and/or bulimia cases. A recent meta-analytic study^31^ on the risk of ICDs in patients with PD versus controls observed an OR from 0.66 to 17.05 for binge eating. These estimates were obtained with heterogeneous study designs, sample sizes, diagnostic criteria and ascertainment methods. In the literature, EDs (e.g. excessive drive to eat or binge-eating disorder) were described in up to 15% of patients with PD who were treated with a DA, leading to weight gain and sometimes to obesity^32,33^.

### FA and NES in PD subjects

Our study is the first to show that FA is very common in patients with PD (24.5%), far exceeding the rate of the general population (9%)^34^. However, other alterations of eating behavior in the population with PD have been already described as “insatiable craving” “with uncontrollable traits” but they have not yet been estimated^35–38^. Based on recent systematic reviews by Pursey et al.^15^ and Burrows et al.^39^ it is estimated that FA affects approximately 20% of the population (range 5.4–56.8%) but the great majority of studies have been performed outside Europe and concern specific populations such as patients attending bariatric surgery or obese subjects.

In our study, 7.0% of patients with PD had NES, which seems to be close to the observed prevalence of nocturnal eating in a prospective study^40^. However, in that study the patients observed underwent deep brain stimulation and therefore were at a more advanced stage of disease compared to our disease cohort, which corresponded to a symptomatic population with PD that had not reached an advanced stage. NES and sleep-related eating disorders have been frequently reported in patients with idiopathic restless legs syndrome (RLS). Interestingly, PD patients may suffer from RLS and an increased frequency of NES was recently reported in PD with RLS compared to those without^41^. Unfortunately, RLS was not assessed in the present study, and we do not know whether the prevalence of RLS differed between PD patients and HCs. Further studies on ED in PD should take into account this potential association.

### FA in the elderly and women

Few studies have focused on FA in the general population, especially in the elderly. One study^42^ focused exclusively on 342 women over the age of 65 and, according to the DSM-5 criteria, observed a prevalence of all EDs at 3.25%, with 1.68% being BED. Binge eating episodes were reported by 5.6% of women and picking/nibbling by 18.9% of women. These figures are close to ours, although our population was mixed in terms of gender and our FA criteria were absent from their study. Around 1.5% of individuals in the general population can present NES^43^. This syndrome has been more specifically observed in women, and 9.1% of them aged ≥ 55 years old reported getting up during the night to eat in the study by Andersen^44^. Food cravings are extremely common, particularly among women^45^, which seems to be confirmed by our study when looking at the multivariate analysis results for FA for the whole population.

Cravings for specific types of foods, such as sweet or high-fat foods, has been described for women with BED^46^, but we did not observe this in the FA analyses, except for chocolate. Patients with PD also reported frequent cravings not only for chocolate but also for sugar-rich foods.

### Association with ICDs and impulsivity

Overall, patients with PD showed more cases of compulsive buying, they had higher depression and anxiety scores and had depression and anxiety disorders more often compared to the general population, characteristics often described in the literature^1,47^ and still significant when comparing a population with or without FA. However, this specific ICD disappeared in the multivariate analysis, may be because it was more related to the over-representation of the PD group. Regarding the other associated disorders, we observed a close to significantly higher attentional impulsiveness score in the FA group, and this factor was still significant in the multivariate analysis in the subgroup of the population with PD (OR 1.16). Attentional impulsiveness has been defined as “an inability to focus attention or concentrate”, and supports the association between FA and attentional deficits that has already been observed in individuals with obesity^48,49^. This factor was not significantly different between patients with PD and the general population, implying that it was very specific for FA. More surprisingly, non-planning impulsivity (orientation to the present rather than to the future) is a protective factor (OR = 0.90) only for FA in patients with PD. Only one study^50^ observed a potential weakly positive relation between non-planning impulsivity and rigid control of eating behavior. The mean non-planning impulsivity score in patients with PD corresponded to normative values, and it is perhaps not directly related to an “eating decision” but rather “having access to food”. Patients with PD who were able to reduce their non-planning impulsiveness (contrary to increasing their ability to plan) may be better able to buy food for later and then consume it when feeling the urge to eat.

### Factors linked to FA in a pooled population

The investigation of factors that were linked to FA in a pooled population showed that a diagnosis of PD (OR 2.40) and being a woman (OR 1.89) are two major risk factors for FA. Gender specificity has been a constant finding in past research on FA in the general population^15^, while the risk factors for PD have been poorly studied^6^ because they have been mainly explored through the prism of the ICD spectrum when discussed around EDs. Kistner et al.^51^ proposed that the vast majority of EDs in patients with PD (excluding BED) should be interpreted as subthreshold pathological behaviors in order to compensate for low dopaminergic signaling and called it “hypodopaminergic snacking”. In the present study, no difference was found between PD patients with or without FA in dopamine ligand type and dose, although dopamine is a well-known contributor to addiction through its differentiated roles in reinforcement, motivation and self-regulation^52–54^. Interestingly, studies of post-STN stimulation observed that the proportion of patients presenting excessive eating behavior remained high in the follow-up, suggesting that the underlying mechanisms of this specific behavior are complex and need forms of management other than just decreased medication^51,55,56^. On the other hand, we observed that impulsive personality modifications were related to FA. Previous studies have linked impulsive personality changes with alterations in dopaminergic activity and receptor availability^57,58^.

### Limitations

A limitation of the study is that, given the context of recruitment, we did not use a specific scale for FA or Food craving. This may prevent this study from being directly comparable to previous research. In addition, actual dietary intake was not measured. Self‐report measures were used. As a result, this tended to overestimate the prevalence rates^59^, but we used validated self-questionnaires. It would be interesting in future works to include rigorous control of food intakes and use specific scales like YFAS^34^. Moreover, we used questions to assess neuropsychiatric symptoms derived from the ASBPD, which has been validated only in populations with PD. The senses of smell and taste, which are known to influence eating behaviors and can be modified in PD^60^, were not explored in our study and future research could explore how these issues could affect eating behaviors in patients with PD. We could not explore whether or not dopamine treatment is a major risk factor for FA in the PD population because of the specificity of patients included (reference center for patients with advanced disease).

Finally, as multifactorial analyses were used, there was no way to determine causal relationships between significant factors and FA.

Although it seems that being attentive to food addiction in Parkinson’s is not common, it should not be neglected, especially in women. New insights into the understanding of the mechanism of FA remain to be investigated, more specifically in patients with PD, and certain components of impulsivity might be targeted in future intervention programs.
